# Determinants of Facility-Based Childbirth in Indonesia

**DOI:** 10.1155/2019/9694602

**Published:** 2019-06-20

**Authors:** Ferry Efendi, Ani Rihlatun Ni'mah, Setho Hadisuyatmana, Heri Kuswanto, Linlin Lindayani, Sarni Maniar Berliana

**Affiliations:** ^1^Faculty of Nursing, Universitas Airlangga, Surabaya, Indonesia; ^2^Department of Statistics, Institut Teknologi Sepuluh Nopember, Indonesia; ^3^Sekolah Tinggi Ilmu Keperawatan PPNI Jawa Barat, Bandung, Indonesia; ^4^Politeknik Statistika STIS, BPS, Jakarta, Indonesia

## Abstract

**Background:**

Reducing maternal mortality remains a significant challenge in Indonesia, especially for achieving the country's Sustainable Development Goals (SDGs) by 2030. One of the challenges is increasing delivery at healthcare facilities to ensure safe and healthy births. In Indonesia, research on factors affecting women's use of facility-based childbirth services is scarce.

**Objective:**

This study was conducted to identify the determinants of facility-based deliveries in Indonesia.

**Methods:**

This study used data from the Indonesia Demographic and Health Survey of 2012, with a cross-sectional design. An odds ratio with 95% confidence intervals (CI) was employed to outline the independent variables for the determinants, including maternal age and education, place of residence, involvement in decision-making, employment status, economic status, and number of antenatal care visits. The dependent variable in this study was the place of delivery: whether it took place in healthcare or nonhealthcare facilities. The statistical significance was set at p<0.05 using bivariate analysis and binary logistic regression.

**Results:**

This study showed that a high level of education (OR: 3.035, 95% CI: 2.310–3.987), high economic status (OR: 6.691, 95% CI: 5.768–7.761), urban residence (OR: 2.947, 95% CI: 2.730–3.181), working status (OR: 0.853, 95% CI: 0.793–0.918), involvement in decision-making (OR: 0.887, 95% CI: 0.804–0.910), and having more than four visits to antenatal care centers (OR: 1.917, 95% CI: 1.783–2.061) were significant determinants of delivery at healthcare facilities.

**Conclusion:**

Efforts to improve facility-based childbirth in Indonesia must strengthen initiatives that promote women's education, women's autonomy, opportunities for wealth creation, and increased uptake of antenatal care, among others. Any barriers related to maternal healthcare services and cultural factors on the use of health facilities for childbirth in Indonesia require further monitoring and evaluation.

## 1. Introduction

Maternal and infant mortality remains a significant global health concern. From 1990 to 2015, the global maternal mortality ratio (MMR) decreased by about 44%, from an estimated 385 to 216 deaths per 100,000 live births. In Southeast Asia, as of 2015, the MMR stands at 164 deaths per 100,000 live births [1]. Although the maternal mortality rate in Indonesia decreased from 359 deaths per 100,000 live births in 2012 to 305 per 100,000 in 2015 [2], this figure is still far from the SDGs for 2030 (below 70 deaths per 100,000 live births). Tragically, more than half of maternal deaths occur during deliveries [3].

Indonesia has made good progress in the area of maternal healthcare services by the increased percentage of deliveries at health facilities from 20.9% in 1991 to 46.6% in 2007 [4, 5]. The latest data also showed that 45.9% of institutional deliveries took place in private facilities such as private hospitals or clinics or with private doctors or midwives and 17% took place in a public facility such as a government hospital or health center [6]. The Indonesian Ministry of Health set the target of achieving 85% of deliveries at health facilities by the year 2019. Therefore, it is critical to identify factors associated with delivery at health facilities. Furthermore, research dealing with factors affecting women's use of facility-based childbirth services in Indonesia's context is scarce.

The World Health Organization (WHO) reported that the use of healthcare facilities to deliver babies significantly decreases maternal mortality [7]. Nonetheless, in Indonesia, only 55.2% of mothers utilize healthcare facilities such as hospitals (whether state- or privately-run), maternity homes, health centers or auxiliary health centers, or general practitioners and practice midwives to assist with childbirth [8]. The Indonesian government has increased the number of healthcare facilities in recent years, not only in urban areas but also in rural locations, and it has also worked to increase the number of healthcare professionals. Nevertheless, many women continue to deliver outside health facilities or prefer to deliver in their homes [9]. Previous studies have highlighted that sociodemographic factors, obstetric characteristics, and access to healthcare facilities are the primary barriers to the use of healthcare facilities for delivery [10–12]. A study conducted in Ethiopia reported that the use of healthcare facilities was determined by maternal age, level of education, household income, parity, attendance of antenatal care four or more times, and birth preparedness [13]. Another study conducted in 34 countries in sub-Saharan Africa reported that older age at first birth was independently associated with higher use of facilities for delivery [14]. Previous research has showed that individual and community level indicators have a positive impact on institutional delivery service uptake [15, 16]. Studies were undertaken in Indonesia highlighting the important role of midwives [17], family influences [18], sociodemographic factors, and access to health insurance [19] associated with institutional birth delivery. However, in the literature, there is a paucity of explanations on whether such predictors play similar roles in determining the utilization of health facilities for delivery in Indonesia, particularly using national data. Thus, this study was designed with the aim of evaluating the determinants of facility-based childbirth in Indonesia.

The next section of this paper is a method that delineates the research data sources, the analytical methods used, and the variables in the study. The section following this outlines and discusses the results of the research. The final part of this paper is a conclusion that contains policy recommendations for related parties.

## 2. Methods

### 2.1. Data Source

This study employs secondary data from the 2012 Indonesian Demographic Data Survey (IDHS). Even though the 2017 IDHS has finished, the final data is not yet available, so the 2012 IDHS is the best choice at this time. The IDHS is part of the international Demographic and Health Survey (DHS) program conducted by the Inner City Fund (ICF). The 2012 IDHS was carried out by the Central Statistics Agency (BPS), in collaboration with the National Population and Family Planning Board (BKKBN) and the Ministry of Health.

The 2012 IDHS sample selection was performed using stratification and multistage random sampling and carried out in 33 provinces in Indonesia from May to August 2012. Each province was divided into districts and then villages. The sample used in this study was married women of reproductive age (ages 15–49). The 2012 IDHS successfully interviewed 43,852 households.

### 2.2. Procedure

The 2012 IDHS obtained ethical approval from the National Institute for Health Research and Development of the Indonesian Ministry of Health. All respondents' identities were deleted from the data, and the respondents gave written approval for their involvement in the research. Permission to use the 2012 IDHS in this study was obtained from ICF International through its website: https://dhsprogram.com/data/new-user-registration.cfm.

### 2.3. Data Analysis

This study employs three stages of analysis: the first is univariate analysis, which is conducted to come to a description of each variable studied; the second is bivariate analysis, done to examine a significant relationship between the independent and dependent variables using the chi-square test; and the last is a logistic regression analysis, performed to identify the significant factors involved in choosing the place of delivery. All data were analyzed using IBM SPSS version 22.0 for Windows.

The dependent variable in this study was the place of delivery, whether or not it took place in a healthcare facility, such as a hospital, clinic, health center, village health post, delivery post, or maternity hospital. The nonhealthcare facilities included the respondent's home or another's home. The reference category of this variable was delivery that took place in a healthcare facility. The analysis unit in this study was all births in the five years before the survey.

The independent variables in this study were maternal age, maternal education, place of residence, women's involvement in household decision-making, working status, economic status, and number of antenatal care visits. The mother's age was divided into seven age groups of five years to see changes in the behavior of each birth cohort of mothers in choosing the place of delivery. The reference category of this variable was the youngest age group (15–19 years). Likewise, the distribution of education levels intended to determine the differences in the behavior of women with the lowest level of education (no education) up to the highest education (university) in the selection of places of delivery. The division of residential areas can be divided into urban and rural areas, where the determination of an area is urban or rural based on the criteria of access and facilities owned. Women's autonomy was measured by the involvement of women in making decisions about respondent's healthcare, divided into two categories, namely, yes and no. Although the availability of data related to employment status is very limited in the IDHS, we sought to cover women's participation in the labor market as measured by working status, based on the question of whether women had worked at least one hour without interruption in the previous week (yes/no). The variable economic status was measured by ownership of durable goods in households divided into five groups, ranging from the poorest to the richest, with reference categories being the poorest. The last explanatory variable is the frequency of examinations during pregnancy, divided into two categories, namely, less than four visits and four or more than four visits.

### 2.4. Binary Logistic Regression

The logistic regression is applied in this paper, since the dependent variable is categorical. The Omnibus test and the Hosmer-Lemeshow test are used to assess the model significance and the model goodness of fit, respectively [20]. The null hypothesis to assess the overall significance of the model is that all coefficients of the covariates in the model are equal to zero. Meanwhile, the null hypothesis to assess the fitted logistic regression is the model fits. The p value needs to be less than 0.05 to indicate that the model is significant in the Omnibus test and more than 0.05 to indicate that the model is fit in the Hosmer-Lemeshow test.

## 3. Results

In this study, of the 17,401 births during the five years before the survey, 55.2% took place in healthcare facilities and 44.8% took place in nonhealthcare facilities (Table 1). More than half of the births were by women who were aged 25–34 years (53%), had secondary education (52.8%), were living in rural areas (54.7%), were not working for a week before enumeration (51.7%), were involved in decision-making (84.6%), were from middle to lower economic groups (68%), and had more than four ANC visits during pregnancy (57.6%).

The results from the bivariate analysis revealed that age, education level, residence area, economic status, number of ANC visits, employment status, and involvement in decision-making were significantly associated with the use of facilities for childbirth (p<0.05; Table 1). Furthermore, Table 1 shows that the percentage of women who delivered in health facilities was lower in the youngest and oldest age groups. The higher the women's education level, the greater the percentage of deliveries in health facilities. The percentage of births in health facilities is greater for women who live in urban areas, do not work, are involved in decision-making, and carry out pregnancy checks at least four times during pregnancy. Furthermore, the more prosperous a woman's household, the more likely she is to deliver in a health facility.

The logistic regression model used yielded p value of 0.001 for the Omnibus test and p value of 0.056 for the Hosmer Lemeshow test, which means that our model is a satisfactory fit for the data. After adjustment for all other variables included, the age of the women no longer had a significant effect on the use of facilities for childbirth (Table 2). The regression model in Table 2 shows that women who had achieved higher levels of education (odds ratio (OR)=3.035, 95% CI: 2.310–3.987), lived in an urban area (OR=2.947, 95% CI: 2.73–3.181), were employed (OR=0.853, 95% CI: 0.793–0.918), enjoyed a higher economic status (OR=6.691, 95% CI: 5.768–7.761), and visited ANC centers more than four times (OR=1.917, 95% CI: 1.783–2.061) were associated with facility-based childbirth in Indonesia.

## 4. Discussion

The results of this study indicate that women who have achieved higher levels of education are more likely to deliver babies in healthcare facilities than those of lower education backgrounds. This is consistent with previous studies in which mothers with higher education were more likely to deliver in healthcare facilities [13, 21]. Education level was found to be significantly associated with lower-risk health behaviors. Mothers who were more educated were more aware of health-related decision-making, how to access information, and how to better plan for future health [21]. However, 33.8% of the other women in our study had a lower level of education (ranging from not attending school to as high as elementary school), indicating the need for more accessible information promoting safe delivery and the use of health facility services for mothers.

Women who live in urban areas prefer to deliver babies in healthcare facilities. An earlier study by Kenea and Jisha [22] in 2017 reported that delivery in healthcare facilities was dominated by urban mothers, while different studies found that mothers in rural areas preferred to deliver in nonhealthcare facilities or at home with traditional, yet noncertified, attendance [23, 24]. The accessibility and availability of facilities for childbirth could well be factors affecting the decision of where to deliver babies for both urban and rural mothers. Given the potential long distance to healthcare facilities faced by those who live in rural areas, it is more likely that rural-living women would prefer to deliver at home, regardless of the birth attendances' competencies [25]. Thus, it is fundamental to assure the accessibility of healthcare facilities, particularly to promote safe delivery for mothers living in rural areas.

Mothers' working status is also a significant determinant in the decision to choose the place for delivery. This study found that Indonesian women who were working were less likely to give birth in healthcare facilities when compared with their peers who were not working. This is in contrast with previous studies that identified that working mothers were associated with giving birth in health facilities [26, 27]. This could possibly be because the mother's quality of work is not good, in the sense that the work does not provide or add to family income significantly, for example, if the mother is a family worker who is not paid. The mother would not have enough funds to give birth in a health facility that is more expensive than giving birth at home, with the assumption that delivery is carried out by nonmedical personnel who are usually more affordable.

Women with a higher economic status were more likely to choose to deliver babies in healthcare facilities than their poorer counterparts, which is a similar finding to previous studies [28–30]. Women who enjoy higher incomes are more able to access healthcare facilities, even when they entail a high cost, and some of the high-quality delivery units are expensive. Health spending in Indonesia is inequitably distributed as Indonesia's Out-of-Pocket (OOP) spending is about average for its income level [31]. This situation might have been worse before Indonesia implemented Law No. 40/2004 on Universal Health Insurance Coverage (UHIC) in 2014 [32]. Our findings highlight inequality in the use of healthcare facilities for delivery in Indonesia, and this could be directly associated with the extent of national healthcare coverage.

Involvement in decision-making is significantly associated with the decision to use a healthcare facility [33]. However, the findings of the current study do not support the previous research. A study that took place in Ethiopia found that maternal autonomy was not substantially related to the use of maternal health facilities for delivery [34]. A study conducted by Kenea and Jisha in 2017 reported that maternal autonomy in decision-making was not significantly related to the choice of delivery place [22]. In Indonesia, religious and cultural beliefs may influence women's autonomy to make such decisions, given that the patriarchal cultural notion that women should obey their husbands is deeply rooted [18]. This being so, healthcare providers need to help families in their decisions on women's healthcare especially related to deliver in healthcare facilities. The active participation of the husband and the decision to accompany the mother to antenatal care might be substantial factors here and would benefit from further scrutiny. In Table 1, it is shown that the percentage of deliveries in health facilities was greater for women with autonomy in decision-making compared to those without autonomy (55.6% versus 52.9%). However, the logistic regression model resulted in the tendency of women with autonomy to utilize health facilities in labor to be smaller compared to women without autonomy. The change in direction of this relationship occurs because the autonomy variable in the regression model has been controlled by the influence of other explanatory variables, while the association in Table 1 is bivariate without taking into account the influence of other variables. If there is a difference like this, then the relationship based on a regression model is considered.

The number of ANC visits is also associated with facility-based childbirth. Mothers who visited ANC units more than four times during their pregnancy were more likely to choose to deliver in healthcare facilities than their counterparts who did not. This is similar to earlier studies by Anastasi, Borchert [35], and Dahiru and Oche [36], which reported that women who often visited ANC units tended to use healthcare facilities for delivery. Gurung et al. (2018) found that women who visited ANC units less than four times were more likely to deliver at home [24], and although more than 80% of pregnant women in Indonesia attend such units more than four times, cultural and religious factors are influential and need to be considered in any approaches to improving the rate of delivery in healthcare facilities.

This research has some limitations. Due to the nature of cross-sectional surveys, the researchers were able to identify associations but not causal effects. Additionally, our study was based on secondary data analysis of the 2012 DHS ARH survey, which may not be able to explore other determinants of the use of healthcare facilities for delivery such as psychological issues and other social aspects. Furthermore, we did not test any interactions between variables in this study.

## 5. Conclusion

This study revealed that Indonesian women's institutional delivery service utilization needs to be improved. Level of education, economic status, residential location, working status, decision making on mother's healthcare, and number of ANC visits were associated with facility-based childbirth in Indonesia. Taking these findings into consideration, it is recommended that maternal healthcare programs should be expanded and promoted toward uneducated and poor women, especially in rural areas, and culturally appropriate campaigns should be considered. Strategies with a focus on increasing recommended antenatal care visit uptakes would benefit the success of delivery in institutional healthcare facilities. Scaling up facility-based childbirth in Indonesia in terms of the numbers, utilization, and quality remains an inevitable issue in maternal healthcare programs. Promoting intersectoral actions to address all associated factors is recommended and would be a benefit for improving health facility-based delivery in Indonesia. Future research on decision-making processes among individuals and at the family level regarding the choice of institutional-based deliveries may provide detailed information.

## Figures and Tables

**Table 1:** A demographic comparison between mothers who deliver in healthcare facilities and nonhealthcare facilities in Indonesia (n=17,401).

Variables	Place of delivery				*X* ^2^
	Healthcare facilities		Nonhealthcare facilities		
	n	%	n	%	
Age (years)					
15–19	250	45.0	306	55.0	114.190
20–24	1606	49.3	1649	50.7	p=0.001
25–29	2754	55.7	2190	44,3	
30–34	2515	58.8	1765	41.2	
35–39	1719	59.0	1193	41.0	
40–44	656	53.5	571	46.5	
45–49	110	48.5	117	51.5	
Education level					
No education	92	17.0	450	83,.0	1908.509
Primary	1910	35.8	3427	64.2	p=0.001
Secondary	5737	62.5	3443	37.5	
Tertiary	1871	79.9	471	20.1	
Place of residence					
Urban	6090	77.3	1792	22.7	2830.044
Rural	3520	37.0	5999	63.0	p=0.001
Working status					
Yes	4551	54.2	3853	45.8	7.581
No	5059	56.2	3938	43.8	p=0.005
Involvement in decision-					
making					
Yes	8190	55.6	6528	44.4	14.302
No	1420	52.9	1263	47.1	p=0.001
Economic status					
Lowest	1283	24.5	3950	75.5	3748.526
Lower middle	1782	51.5	1677	48.5	p=0.001
Medium	1990	63.4	1151	36.6	
Upper middle	2260	77.0	676	23.0	
Highest	2295	87.2	337	12.8	
ANC visits					
<4	3012	40.8	4362	59.2	1070.251
≥4	6598	65.8	3429	34.2	p=0.001

Total	9610	55.2	7791	44.8	

Note: *∗*p<0.05; *∗∗* p < 0.01; *∗∗∗*p < 0.001.

**Table 2:** The odds ratio of the use of health facilities for delivery in Indonesia (n=17,401).

Variables	Odds ratio	95% CI for EXP(B)	
	(OR)	Lower	Upper
Age (years)			
15–19	-	-	-
20–24	1.004	0.818	1.232
25–29	1.013	0.829	1.239
30–34	1.042	0.850	1.278
35–39	1.227	0.995	1.514
40–44	1.008	0.860	1.375
45–49	1.080	0.745	1.564
Education level			
No education	-	-	-
Elementary	1.348*∗*	1.050	1.731
Secondary	2.263*∗∗∗*	1.763	2.904
Tertiary	3.035*∗∗∗*	2.310	3.987
Place of residence			
Rural	-	-	-
Urban	2.947*∗∗∗*	2.730	3.181
Working status			
No	-	-	-
Yes	0.853*∗∗∗*	0.793	0.918
Involvement in decision-making			
No	-	-	-
Yes	0.887*∗*	0.804	0.910
Economic status			
Lowest	-	-	-
Lower middle	2.171*∗∗∗*	1.967	2.395
Medium	2.747*∗∗∗*	2.472	3.053
Upper middle	4.275*∗∗∗*	3.796	4.814
Highest	6.691*∗∗∗*	5.768	7.761
ANC visits			
<4	-	-	-
≥4	1.917*∗∗∗*	1.783	2.061

Note: *∗* p< 0.05; *∗∗* p < 0.01; *∗∗∗*p < 0.001.

## Data Availability

The data used to support the findings of this study are available from the corresponding author upon request.

## References

[1] Bongaarts J. (2016). WHO, UNICEF, UNFPA, World Bank Group, and United Nations Population Division trends in maternal mortality: 1990 to 2015 Geneva: World Health Organization, 2015. *Population and Development Review*.

[2] MoH (2016). *Laporan Tahunan Kesehatan Keluarga*.

[3] Balitbangkes-MoH (2012). *Disparitas, Akses dan Kualitas. Kajian Determinan Kematian Maternal di Lima Region Indonesia (Disparity, Access and Quality. A study on Maternal Death Determinants in Five Regions of Indonesia)*.

[4] Statistics Indonesia (2008). *Indonesia Demographic and Health Survey 2007*.

[5] Statistics Indonesia (1992). *Indonesia Demographic and Health Survey 1991*.

[6] Statistics Indonesia (2013). *Indonesia Demographic and Health Survey 2012*.

[7] WHO (2019). *Maternal mortality*.

[8] Tejayanti T., Perwitasari D., Sulistyowati N., Trihono (2014). *Kajian Layanan Kesehatan Ibu (Review of Maternal Health Service)*.

[9] MoH (2017). *Profil Kesehatan Indonesia 2017 [Indonesia Health Profile 2017]*.

[10] Tsegay Y., Gebrehiwot T., Goicolea I., Edin K., Lemma H., Sebastian M. S. (2013). Determinants of antenatal and delivery care utilization in Tigray region, Ethiopia: a cross-sectional study. *International Journal for Equity in Health*.

[11] Yesuf E. A., Kerie M. W., Calderon-Margalit R. (2014). Birth in a health facility -inequalities among the Ethiopian women: results from repeated national surveys. *PLoS ONE*.

[12] Birmeta K., Dibaba Y., Woldeyohannes D. (2013). Determinants of maternal health care utilization in Holeta town, central Ethiopia. *BMC Health Services Research*.

[13] Asseffa N. A., Bukola F., Ayodele A. (2016). Determinants of use of health facility for childbirth in rural Hadiya zone, Southern Ethiopia. *BMC Pregnancy and Childbirth*.

[14] Dunlop C. L., Benova L., Campbell O. (2018). Effect of maternal age on facility-based delivery: analysis of first-order births in 34 countries of sub-Saharan Africa using demographic and health survey data. *BMJ Open*.

[15] Mekonnen Z. A., Lerebo W. T., Gebrehiwot T. G., Abadura S. A. (2015). Multilevel analysis of individual and community level factors associated with institutional delivery in Ethiopia. *BMC Research Notes*.

[16] Dickson K. S., Adde K. S., Amu H. (2016). What influences where they give birth? determinants of place of delivery among women in rural Ghana. *International Journal of Reproductive Medicine*.

[17] Kartikarini A. (2012). *Determinan Pemilihan Tempat Persalinan di Indonesia Timur (Analisis Data IFLS East 2012)*.

[18] Asfian P. (2016). Determinants of mother’s choice of place delivery in community of Bajo, Muna district: a qualitative study. *Public Health of Indonesia*.

[19] Arief M., Sudikno S. (2010). Determinan pemilihan persalinan di fasilitas kesehatan (analisis data riset kesehatan dasar tahun 2010). *Indonesian Journal of Reproductive Health*.

[20] Hosmer D. W., Lemeshow S. (2013). *Applied Logistic Regression*.

[21] Shahabuddin A. S. M., De Brouwere V., Adhikari R., Delamou A., Bardaj A., Delvaux T. (2017). Determinants of institutional delivery among young married women in Nepal: evidence from the Nepal Demographic and Health Survey, 2011. *BMJ Open*.

[22] Kenea D., Jisha H. (2017). Urban-rural disparity and determinants of delivery care utilization in Oromia region, Ethiopia: community-based cross-sectional study. *International Journal of Nursing Practice*.

[23] Javed S. A., Anjum M. D., Imran W. (2013). Correlates of preferences for home or hospital confinement in Pakistan: evidence from a national survey. *BMC Pregnancy and Childbirth*.

[24] Gurung M. S., Pelzom D., Wangdi S., Tshomo T., Lethro P., Dema T. (2018). Factors associated with delivery at home in Bhutan: findings from the National Health Survey 2012. *WHO South-East Asia Journal of Public Health*.

[25] Yaya S., Bishwajit G., Uthman O. A., Amouzou A., Leone T. (2018). Why some women fail to give birth at health facilities: a comparative study between Ethiopia and Nigeria. *PLoS ONE*.

[26] Ngowi A. F., Kamazima S. R., Kibusi S., Gesase A., Bali T. (2017). Women’s determinant factors for preferred place of delivery in Dodoma region Tanzania: a cross sectional study. *Reproductive Health*.

[27] Fekadu G. A., Ambaw F., Kidanie S. A. (2019). Facility delivery and postnatal care services use among mothers who attended four or more antenatal care visits in Ethiopia: further analysis of the 2016 demographic and health survey. *BMC Pregnancy and Childbirth*.

[28] Roro M. A., Hassen E. M., Lemma A. M., Gebreyesus S. H., Afework M. F. (2014). Why do women not deliver in health facilities: a qualitative study of the community perspectives in south central Ethiopia?. *BMC Research Notes*.

[29] Caulfield T., Onyo P., Byrne A. (2016). Factors influencing place of delivery for pastoralist women in Kenya: a qualitative study. *BMC Women's Health*.

[30] Do M., Soelaeman R., Hotchkiss D. R. (2015). Explaining inequity in the use of institutional delivery services in selected countries. *Maternal and Child Health Journal*.

[31] The World Bank (2010). *Indonesia’s Health Sector Review*.

[32] Mboi N. (2015). Indonesia: on the way to universal health care. *Health Systems & Reform*.

[33] Kifle M. M., Kesete H. F., Gaim H. T. (2018). Health facility or home delivery? Factors influencing the choice of delivery place among mothers living in rural communities of Eritrea. *Journal of Health, Population and Nutrition*.

[34] Kebede A., Hassen K., Teklehaymanot A. N. (2016). Factors associated with institutional delivery service utilization in Ethiopia. *International Journal of Women's Health*.

[35] Anastasi E., Borchert M., Campbell O. M. R. (2015). Losing women along the path to safe motherhood: Why is there such a gap between women's use of antenatal care and skilled birth attendance? A mixed methods study in northern Uganda. *BMC Pregnancy and Childbirth*.

[36] Dahiru T., Oche O. M. (2015). Determinants of antenatal care, institutional delivery and postnatal care services utilization in Nigeria. *Pan African Medical Journal*.

